# Bioinspired Adaptive, Elastic, and Conductive Graphene Structured Thin-Films Achieving High-Efficiency Underwater Detection and Vibration Perception

**DOI:** 10.1007/s40820-022-00799-4

**Published:** 2022-02-15

**Authors:** Qiling Wang, Peng Xiao, Wei Zhou, Yun Liang, Guangqiang Yin, Qiu Yang, Shiao-Wei Kuo, Tao Chen

**Affiliations:** 1 Key Laboratory of Marine Materials and Related Technologies, Zhejiang Key Laboratory of Marine Materials and Protective Technologies, Ningbo Institute of Materials Technology and Engineering, Chinese Academy of Sciences, Zhongguan West Road 1219, Ningbo, 315201 People’s Republic of China; 2 School of Chemical Sciences, University of Chinese Academy of Sciences, 19A Yuquan Road, Beijing, 100049 People’s Republic of China; 3 Ningbo New Material Testing and Evaluation Center Co., Ltd, Ningbo, 315000 People’s Republic of China; 4 Department of Material and Optoelectronic Science, Center of Crystal Research, National Sun Yat-Sen University, Kaohsiung, 804 Taiwan, People’s Republic of China

**Keywords:** Janus film, Water depth detection, Vibration perception

## Abstract

Underwater exploration has been an attractive topic for understanding the very nature of the lakes and even deep oceans. In recent years, extensive efforts have been devoted to developing functional materials and their integrated devices for underwater information capturing. However, there still remains a great challenge for water depth detection and vibration monitoring in a high-efficient, controllable, and scalable way. Inspired by the lateral line of fish that can sensitively sense the water depth and environmental stimuli, an ultrathin, elastic, and adaptive underwater sensor based on Ecoflex matrix with embedded assembled graphene sheets is fabricated. The graphene structured thin film is endowed with favourable adaptive and morphable features, which can conformally adhere to the structural surface and transform to a bulged state driven by water pressure. Owing to the introduction of the graphene-based layer, the integrated sensing system can actively detect the water depth with a wide range of 0.3–1.8 m. Furthermore, similar to the fish, the mechanical stimuli from land (e.g. knocking, stomping) and water (e.g. wind blowing, raining, fishing) can also be sensitively captured in real time. This graphene structured thin-film system is expected to demonstrate significant potentials in underwater monitoring, communication, and risk avoidance.

**Supplementary Information:**

The online version contains supplementary material available at 10.1007/s40820-022-00799-4.

## Highlights

- The lateral-line-like underwater mechanical sensor (LUMS) realizes the imitation of the structure and function of the lateral line.
- The detection range of water depth can be controlled by adjusting the size of the graphene/Ecoflex Janus film on LUMS. The maximum measured depth is 1.8 m.
- Similar to the fish, the mechanical stimuli from land and water can be sensitively captured by LUMS in real time.

**Supplementary Information:**

The online version contains supplementary material available at 10.1007/s40820-022-00799-4.

## Graphical abstract

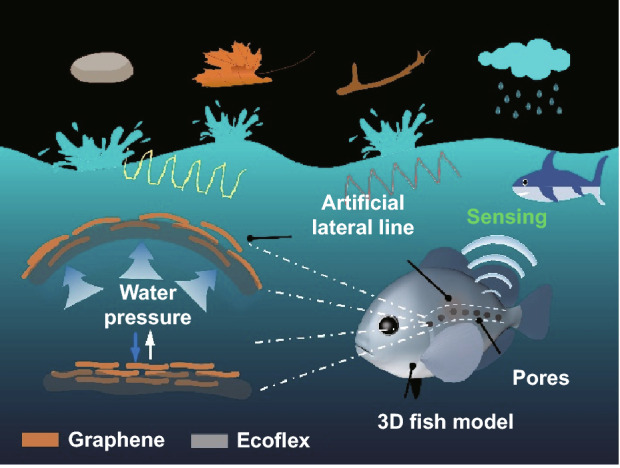

## Introduction

Exploration in extreme environments, especially underwater monitoring and communication, is of great significance to understand the unknown and/or unmapped underwater world. Precisely and stably capturing underwater signal of depth and dramatic/tiny vibration can provide abundant and essential information for underwater forewarning, creature tracking, and environmental considerations [1–6]. To date, extensive efforts have been devoted to developing underwater sensing materials and their integrated devices [7–12]. Soft materials featured with anti-corrosion and flexibility are considered as a promising candidate, enabling piezoresistive [13–15], capacitive [16, 17] and piezoelectric [7, 18] mechanism for underwater sensing. Since the water environment may adversely affect the conductivity of the sensors, some strategies have been developed to tackle this problem, including superhydrophobic sealing [19–23], and polymer encapsulation [24–26]. Among them, the superhydrophobic sealing method heavily relies on the micro-nano-structures [15, 19], where the long-term stability may experience unfavourable wettability failure. And the encapsulation one may lose sensitivity of devices. In addition, eye-catching forms of materials and integrated devices may be easily attacked by underwater creatures, which should also be considered. Thus, the design of underwater sensors with flexible, environmentally stable and imperceptible properties for broad water depth detection and tiny vibration perception still remains a challenge in one integrated system.

**Fig. 1:**
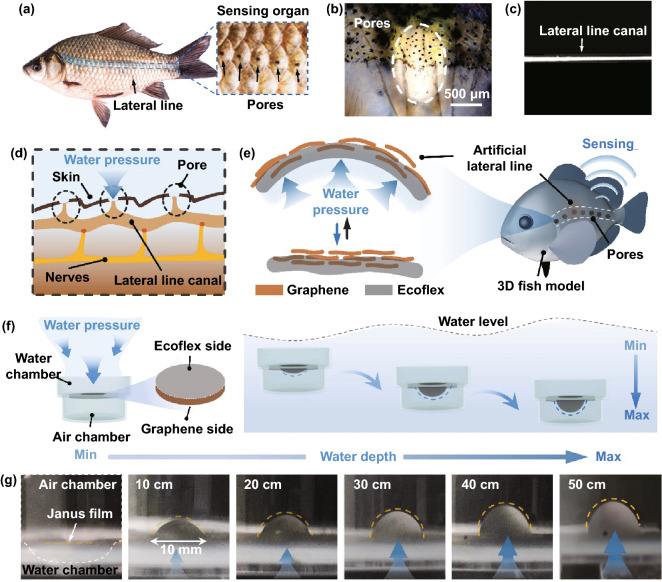
**a** The lateral line perception system of fish. **b** The microscopic image of fish scales. **c** The digital photograph of lateral line canal. **d** The schematic diagram of a lateral line system. **e** Schematic illustration of the lateral line. **f**, **g** The digital photographs of the graphene/Ecoflex Janus film under water at depths of 0, 10, 20, 30, 40, and 50 cm, respectively

In nature, fish can actively sense the external environment (e.g. water depth and mechanical vibration) and further adapt their behaviours to the surroundings. In the fish sensing systems, the lateral line has played vital roles in perceiving the external water pressure, flow movement and diverse vibrations (Fig. 1a-c). In water, the pressure change can go through the pores on the scales to the lateral line canal, which can act on the nerves for real-time and high-sensitive underwater sensing (Fig. 1d) [27–31]. Inspired by the fish sensing system, herein, we developed an artificial lateral line system enabled by a flexible and imperceptible thin-film in a self-supported state to simultaneously perceive wide-range underwater depth and large/tiny vibration. To achieve stable underwater sensing, a Janus film structure was designed and further integrated as a self-supported sensor with Ecoflex layer exposed to water. As a proof of concept, an artificial fish lateral line system was constructed to imitate the underwater sensing functions of fish. Owing to the ultrathin and elastic features, the fabricated film-based sensor can experience a remarkable deformation to achieve reversible resistance change for real-time water depth detection with a maximum depth of 1.8 m. Moreover, similar to fish, it can sensitively perceive the tiny vibration above and below the water surface, such as wind blowing, raining, underwater creatures’ attacking, etc. The novel and simple design of this work is expected to demonstrate significant potentials in applications of underwater monitoring, communication and rescue.

## Materials and Methods

### Materials

Graphene slurry (5 wt%) with surface defects (Supplementary Fig. S1) obtained by mechanical exfoliation was acquired from Ningbo Morsh Technology Co., Ltd, and the size range of graphene flakes was ~ 2.5–7 μm (Supplementary Fig. S2). Owing to the multi-layered structure, the thickness of graphene flakes was ~ 10 nm (Supplementary Fig. S3). Silicon rubber (Ecoflex™ 00-50) was obtained from Smooth-On, Inc. N-heptane (AR, 98%) was acquired from Shanghai Aladdin Biochemical Technology Co., Ltd. Ethyl alcohol (AR, ≥ 99.7%) was purchased from Sinopharm Chemical Reagent Co., Ltd. Deionized water was used as the substrate to prepare single graphene film and graphene/Ecoflex Janus film at the air/water interface.

### Fabrication of Graphene Film

Firstly, graphene slurry (5 g) was dispersed in 250 mL anhydrous ethanol, followed by strong ultrasonication (250 W) for about 6 h to obtain a stable dispersion. Then, the graphene dispersion (35 mL) was sprayed equably on the water surface. After stabilization for about 30 min, the microporous sponge was put on one side of the water/air interface to siphon water, while the graphene nanosheets were tightly stacked toward the opposite direction of the siphon. Finally, the graphene film with a closely packed structure was formed at the water/air interface until the pre-assembled film could not be compressed further.

### Fabrication of Graphene/Ecoflex Janus Film

The Ecoflex part A and part B (1:1, w/w) were dissolved in n-heptane with a weight ratio of 7.71% and then ultrasonicated for 5 min to obtain a homogeneous solution. After that, the Ecoflex solution (35 mL) was dropwise added onto the graphene/air interface along the container wall, followed by the evaporation of n-heptane and curing of Ecoflex at room temperature for 6 h. Subsequently, the graphene/Ecoflex ultrathin Janus hybrid film was obtained at the water/air interface.

### Preparation of Lateral-Line-Like Underwater Mechanical Sensor

At first, a hole with an appropriate diameter was cut out in the middle of the petri dish, and then an ultrathin layer of Ecoflex prepolymer was scraped on its surface after rinsing the petri dish with anhydrous ethanol. The prepared graphene/Ecoflex Janus film was transferred onto the surface of the petri dish with part of the film attached to the substrate and the other part self-supported. After the curing of Ecoflex prepolymer layer at 60 °C for 1 h, the film was closely attached to the substrate. Then, the aluminium wire was connected to the graphene side with the aid of silver paste curing in a 60 °C oven. After that, another polymethyl methacrylate (PMMA) lid (diameter: 36 mm, height: 15 mm) was covered onto the graphene side and sealed with Ecoflex. Finally, the lateral-line-like underwater mechanical sensor, whose graphene sensing layer was encapsulated and Ecoflex elastic layer was exposed to water, was successfully fabricated.

### Characterization and Measurements

Surface and cross section morphology of the film was observed by Hitachi-S4800 field-emission scanning electron microscope (FE-SEM) at an accelerating voltage of 4 kV. The microscopic images of fish scales and tapes were captured by an OLYMPUS BX51 polarizing microscope. The Raman scattering measurements were carried out by an R-3000HR spectrometer (Raman Systems, Inc., R-3000 series) excited by a solid-state diode laser (532 nm), with a frequency range of 3500–200 cm^−1^. Z1 Zwick/Roell Universal Testing System was used to test the stress–strain characteristics of films. When LUMS detected underwater depth or external stimuli, the electrodes at both ends of the sensor were connected to the electrochemical workstation through wires, and the electrochemical workstation was connected to the computer (Supplementary Fig. S4). Electrochemical Workstation (CH Instruments, CHI660E.Chenhua Co., Shanghai, China) was used to record the real-time current (*I*) accompanied by a constant voltage (*V*_0_) of 1 V, while the real-time resistance (*R*) was calculated by the equation *R* = *V*_0_/*I*. The water contact angle was measured using Dataphysics OCA25 instrument by carefully dropping a 3-µL water droplet on the surface of film. AFM measurements were conducted using Dimension ICON SPM (Bruker, USA) in a Peak Force tapping mode.

## Results and Discussion

### Fabrication and Application of the Janus Films

Inspired by the structure and sensing mechanism of the lateral line, a Janus film composed of graphene and Ecoflex was designed via interfacial functionalization strategy. The assembled graphene film functioned as a sensing layer of the Janus film. It was fabricated via Marangoni effect induced self-assembly and the capillary force driving compression at the air/water interface [32–34]. And the thickness of the assembled graphene film was about 200 nm (Supplementary Fig. S5). Subsequently, Ecoflex prepolymer elastomer dispersed in heptane solution was gradually dropped onto the surface of graphene film, followed by a curing procedure. The interfacial functionalization strategy enables the formation of ultrathin Janus film at the air/water interface (Supplementary Fig. S6a), and the initial resistance of the Janus film (59.5 μm × 1.5 cm × 3.9 cm) was 6.98 kΩ. In order to explore the hydrophilicity and hydrophobicity of the Janus film, water contact angle was measured by carefully dropping a 3-µL water droplet on the surface of films. In comparison with the pure graphene film with a water contact angle of 20.2°, the graphene side of the Janus film displayed a bigger water contact angle of 88.9° (Supplementary Fig. S7). In addition, the water contact angle of Ecoflex side of the Janus film is 109.4°, similar to that of the pure Ecoflex film (Supplementary Fig. S7). The hydrophobic property of Ecoflex layer ensures the hydrophobicity of the Janus film and makes it possible to be used underwater.

Owing to the ultrathin and conductive features of the Janus film, it can be driven to deform by tiny water vibration and low/high water pressure for real-time electrical signal output. As an analogue to the lateral line of fish, it can actively detect the mechanical stimuli from the surrounding environments and sense the change of water pressure decided by the water depth (Fig. 1e). Therefore, by mimicking the structure of the real lateral line, the acquired Janus film can be conformally transferred onto a model fish to achieve a lateral-line-like underwater mechanical sensor (LUMS). In our system, a model with a hollow structure was designed to assemble with the Janus film for a self-supported structure, in which the Ecoflex layer was exposed to water and the graphene layer was sealed in the air chamber of the model. Besides, the electrodes were applied on the surface of the graphene layer to form an LUMS (Fig. 1f). With the increase in water depth, the Janus film can experience a simultaneous deformation, accompanied by a corresponding current change for real-time depth detection. As shown in Fig. 1g, LUMS was located at a series of water depths, such as 0, 10, 20, 30, and 40 cm. It can be clearly observed that the film can be actuated by the water pressure and actively experience a gradual increase in the degree of deformation. The real-time mechanical deformation is expected to induce the corresponding electrical signal change for efficient water depth detection.

### Structural Characterization and Properties of the Janus Film

**Fig. 2:**
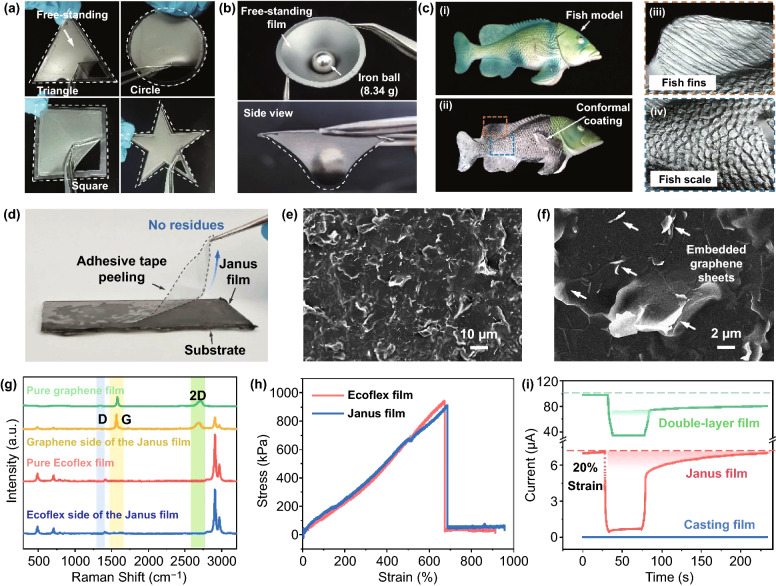
**a** The digital photographs of self-supported graphene/Ecoflex Janus film on frames with different shapes. **b** The digital photographs of the graphene/Ecoflex Janus film holding an iron ball with a weight of 8.34 g. **c** The digital photographs of the graphene/Ecoflex Janus film attached to the surface of the model fish. **d** Peeling-off of the adhesive tape from the graphene side of the Janus film. **e**, **f** SEM images of the graphene side surface of the graphene/Ecoflex Janus film. **g** Raman spectra of the pure Ecoflex film, pure graphene film and both sides of the graphene/Ecoflex Janus film. **h** Representative tensile stress–strain curves of the pure Ecoflex film and Janus film. **i** Current of three kinds of films before and after applying 20% tensile strain

Since the interface functionalization strategy can enable the formation of an ultrathin and transferrable film, the achieved Janus film can be easily transferred onto diverse targets for conformal and robust adhesion. As shown in Fig. 2a, hollow models with different shapes were employed to assemble with the Janus film for a self-supported one. It is observed that the film can adapt smoothly to various sophisticated shapes (e.g. triangle, circle, square, and pentacle). When it functioned as a self-supported one, the film can even support objects such as an iron ball with a weight of 8.34 g. The result suggests that the film can demonstrate robust mechanical strength (Fig. 2b). In addition, the ultrathin Janus film also shows excellent conformal characteristic, which can adapt conformally to complex surfaces with complicated embossment. Here, a fish model with fine structure of scales and fins was used. Figure 2c represents that the film can spread smoothly along the structured embossment of the fish model, in which the scales and fins can be entirely and clearly observed. Similarly, models such as starfish and seaweed were also selected as targeted substrates, demonstrating a good conformal ability of the film (Supplementary Figs. S8 and S9). Moreover, the interface functionalization method also allows the formation of stable interface between graphene and Ecoflex layer. As shown in Fig. 2d, the adhesive tape was tightly adhered onto the graphene side of the film, followed by a peeling-off operation without visible residues. As shown in Supplementary Fig. S10, although there are some black residues on the surface of the tape, they are unevenly distributed and stand for bits of weak-bonded graphene on the surface. And most of the graphene is embedded in Ecoflex and stays stable on the film.

In order to evidence the advantage of this interface functionalization method, two control samples were fabricated in our experiments, including the conventional casting method (Supplementary Fig. S6b) and transferring method (Supplementary Fig. S6c). In detail, for the conventional casting method, the assembled graphene film at the water/air interface was transferred onto the glass surface, followed by casting Ecoflex prepolymer solution on its surface. However, the interaction between the graphene film and the glass substrate is too strong to allow for intact peeling-off of the double-layer film (Supplementary Fig. S11a). For the transferring one, a pure Ecoflex film was firstly fabricated at the water/air interface, which was further cut and transferred onto the glass substrate. Then, the prepared graphene film was transferred and covered on the Ecoflex film at the air/water interface. After natural air-drying procedure, the double-layered film was obtained. The results show that the curing Ecoflex film cannot effectively interact with the graphene nanosheets for a Janus film due to the weak penetration to the closely packed sheets. Sample of the transferring method presents poor stability of the graphene layer, which can be easily peeled off using the adhesive tape (Supplementary Fig. S11b). Generally, compared to the conventional casting method and transferring method, the Janus film fabricated by the interface functionalization method demonstrates a stable interface between the graphene layer and the Ecoflex layer. Furthermore, SEM characterization was also conducted to investigate the micro-scale morphology of the Janus film. Figure 2e, f and Supplementary Fig. S12 clearly show that the graphene nanosheets are partially embedded into the elastomeric matrix and the exposed graphene sheets can provide contact sites for the formation of a conductive pathway. And there was only a wrinkled structure of elastomer on the Ecoflex side (Supplementary Fig. S13).

To further investigate the structural information of the Janus film, Raman spectra was also used to characterize the asymmetric structure. As illustrated in Fig. 2g, the result reveals that the Ecoflex side of Janus film has the same Raman curve as the pure Ecoflex, and the characteristic peak location is mainly in the low frequency (490 cm^−1^, 710 cm^−1^) and high frequency (2906 cm^−1^, 2965 cm^−1^) regions. For the graphene side, it not only has the same D (1348 cm^−1^), G (1579 cm^−1^) and 2D (2718 cm^−1^) characteristic peaks as the pure graphene film, but also has the same characteristic peaks as the pure Ecoflex. The result further indicates that the side of the graphene layer is partially wrapped by Ecoflex to form a semi-embedded structure, which may result from the infiltration of Ecoflex solution into the graphene layer during the preparation process. In addition, the Raman mapping can also clearly illustrate the Janus structure of this film (Supplementary Fig. S14). More importantly, compared with the pure Ecoflex film, the asymmetric introduction of the graphene layer into the elastomer system may not remarkably affect the mechanical strength (Fig. 2h). Additionally, the electrical information of three samples was also measured in Fig. 2i. When a 20% strain was applied and then released on the samples, only the current of Janus film can recover to the initial value, representing good electrical stability and repeatability (Supplementary Fig. S15).

Moreover, the strain sensing performance of the Janus film-based strain sensor was investigated, including sensitivity, stability as well as the responses to different strains and stretching frequencies, which was represented by a relative resistance change (Δ*R*/*R*_0_):

1 $$\Delta {/}R_{0} = \left( {R - R_{0} } \right){/}R_{0}$$

where *R* is the instantaneous resistance at the stretched state and *R*_0_ is the initial resistance at the relaxed state. The tensile strain (*ε*) and Gauge factor (GF) are calculated by Eqs. (2) and (3), respectively:

2 $$\varepsilon = \left( {L - L_{0} } \right)/L_{0}$$

3 $${\text{GF}} = \frac{{\delta \left( {\Delta R{/}R_{0} } \right)}}{\delta \varepsilon }$$

**Fig. 3:**
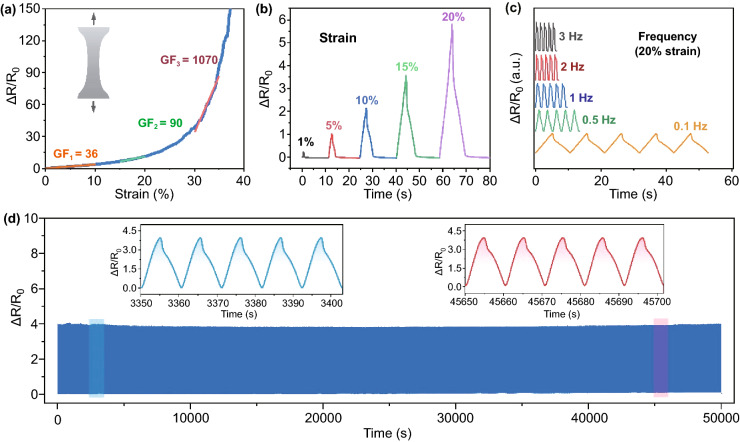
**a** The sensing performance of the graphene/Ecoflex Janus film and specific GF values under different tensile strains. Δ*R*/*R*_0_ versus time curve of the Janus film **b** under various tensile strains and a stretching velocity of 50 mm min^−1^; **c** under a tensile strain of 20% and various frequencies of 0.1, 0.5, 1, 2, and 3 Hz; **d** under a tensile strain of 20% for 5000 cycles

As shown in Supplementary Fig. S16, with the increase in the concentration of the graphene dispersion, the response of tensile strain range of the Janus film expanded. And the sensitivity of the Janus film gradually decreased in the same tensile strain range. In order to ensure the excellent conductivity and high sensitivity of the Janus film under large deformation, graphene dispersion (1 mg mL^−1^) was chosen to prepare the assembled graphene film and the Janus film. As shown in Fig. 3a, the Janus film-based strain sensor displays a large GF in the full sensing range. The corresponding GFs for the sensor are 36 (*ɛ*: 0–10%), 90 (*ɛ*: 15–20%), and 1070 (*ɛ*: 30–35%), respectively, which demonstrate that the Janus film-based strain sensor has the potential ability to sense external stimuli with high sensitivity. The relative resistance increases with the increasing strain under cyclic tensile strain of 1%, 5%, 10%, 15%, and 20% (Fig. 3b), which shows good resolution at each different strain. The stability of the Janus film was also studied. As shown in Fig. 3c, the relative resistance change is almost independent of the frequency with a tensile strain of 20% within a range of 0.1–3 Hz. Besides, the relative resistance change varies periodically and no obvious fluctuation or drift occurs in each cycle, presenting excellent stability and long-term durability during cyclic tensile tests between 0 and 20% tensile strain for 5000 cycles (Fig. 3d).

### Water Depth Detection of the LUMS

Based on the sensitive and stable electrical performance of the Janus film, it was further integrated into LUMS to achieve water depth detection. The sensitivity of the conductive material might be negatively affected when assembled into underwater sensors fabricated by polymer encapsulation method. During the polymer encapsulation, the polymer penetrates into the gap of the conductive material, resulting in an increase in the initial resistance of the conductive material. As shown in Supplementary Fig. S17, compared with the sensor achieved by the Janus film encapsulated by Ecoflex, LUMS based on the Janus film possesses good sensing sensitivity and electrical stability. As displayed in Supplementary Fig. S18, a specific model with a hollow circle structure was designed and the Janus film was subsequently transferred onto the model surface with integrated electrodes. Similar to the lateral line of the fish that can sense the water pressure mediated by the pores on the scales, LUMS allows the Ecoflex side exposed to water and the graphene side encapsulated in the chamber. As a result, with the increase in water depth, the generated water pressure can drive the film to deform to a balanced state. Note that the water pressure (*P*_1_) applied on the Janus film was calculated according to Eq. (4):

4 $$P_{1} = \rho gh$$

The relationship between gas pressure (*P*_2_) and volume (*V*) in the covered seal system can be as follows:

5 $$P_{2} V = nRT$$

where *ρ* represents the density of water, *g* is the acceleration of gravity, and *h* is the height from LUMS to the water surface, n is the amount of gas substance, *T* is the absolute temperature, and *R* is a constant of about 8.314 J K^−1^ mol^−1^. When LUMS was employed to detect water depth, the film experienced inward deformation driven by the water pressure. The film deformation can prominently reduce the volume of the air chamber to further increase the inner air pressure. When *P*_1_ was in equilibrium with *P*_2_, the dynamic deformation of the film achieved an equilibrium state.

**Fig. 4:**
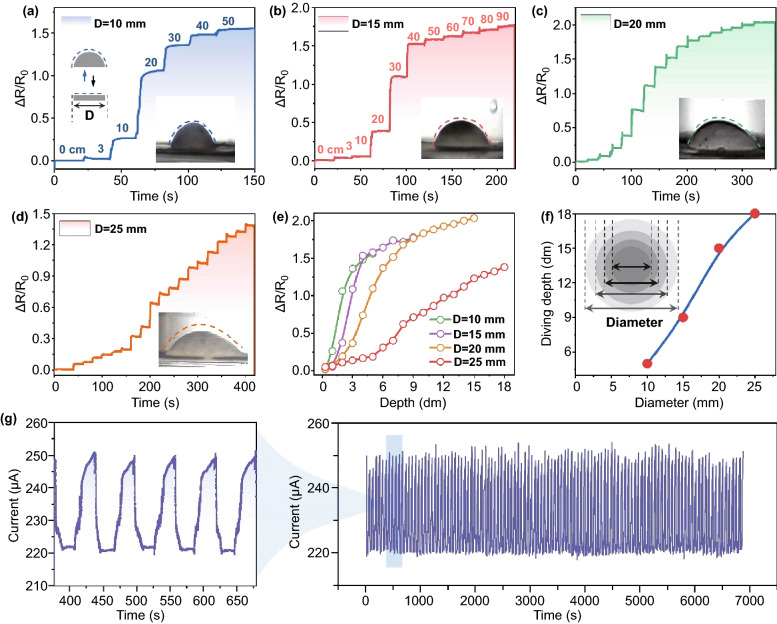
Δ*R*/*R*_0_ versus time curve of LUMS at different depths with the diameter of **a** 10 mm, **b** 15 mm, **c** 20 mm, and **d** 25 mm, and digital photographs of the Janus film inside LUMS at corresponding maximum measurable depth. **e** Δ*R*/*R*_0_ versus depth curves of LUMS with different diameters. **f** The curve of the maximum measurable depth as a function of the film diameters. **g** Output current versus time curve where LUMS went back and forth between the liquid level and the maximum measurable depth

In our system, with the increase in the water depth, there appeared apparent film deformation and simultaneous terraced current platform located at a certain depth. Take the sample of the self-supported film with a diameter of 10 mm as an example, LUMS can respond sensitively to the depth of 3 cm and detect up to the depth of 50 cm (Fig. 4a). When further increasing the depth to 60 cm, the resulted current no more changed and the resultant maximum depth was finally achieved (Supplementary Fig. S19). More interestingly, it is found that the degree of deformation at the same depth is also related to the diameter of the self-supported film. When increasing the diameter of the hollow circle, the degree of deformation may decrease gradually. Thus, devices with four kinds of diameter were designed, including 10, 15, 20, and 25 mm. As illustrated in Fig. 4b–d, all the three samples with certain diameters demonstrate step-like change in current with the increase in water depth. It is found that the maximum detection depth is positively related to the diameter. For example, the sample with a diameter of 25 mm can realize a good electrical response at a depth up to 1.8 m. However, owing to the decreased film deformation at a certain depth, the relative resistance of these samples experienced a reduced tendency at the same depth with the increase in diameters (Fig. 4e). Furthermore, Fig. 4f and Supplementary Fig. S20 clearly show the positive linear relationship between diameter and maximum water depth.

To further explore the influence of film diameter on sensing performance of LUMS, the response time of sensors of different sizes to the same stimulus was detected. The response time is defined as the time required to reach a relative resistance variation that is equal to 90% of the peak value. As shown in Supplementary Fig. S21, the response time of the sensor with a film diameter of 10, 15, 20, and 25 mm was 0.032, 0.046, 0.050, and 0.076 s, respectively. In general, the response time of the sensor increases with the increase of the film diameter. Moreover, repeatability test was also conducted, where the device was moved back and forth between the liquid level and the maximum measurable depth and demonstrated good stability of the electrical performance (Fig. 4g). Considering that LUMS was used for underwater sensing, the influence of water temperature on the sensor's sensing performance was studied. As shown in Supplementary Fig. S22, when the temperature suddenly increased by 3 °C, the relative resistance change kept increasing, and it still could not reach a stable state even after 200 s, which differed greatly from the response time when LUMS was subjected to external stimuli. Therefore, when detecting water depth or perceiving external stimuli, the effect of the water temperature change on the sensing performance of LUMS was almost negligible.

### Multifunctional LUMS for Underwater and Waterside Monitoring

**Fig. 5:**
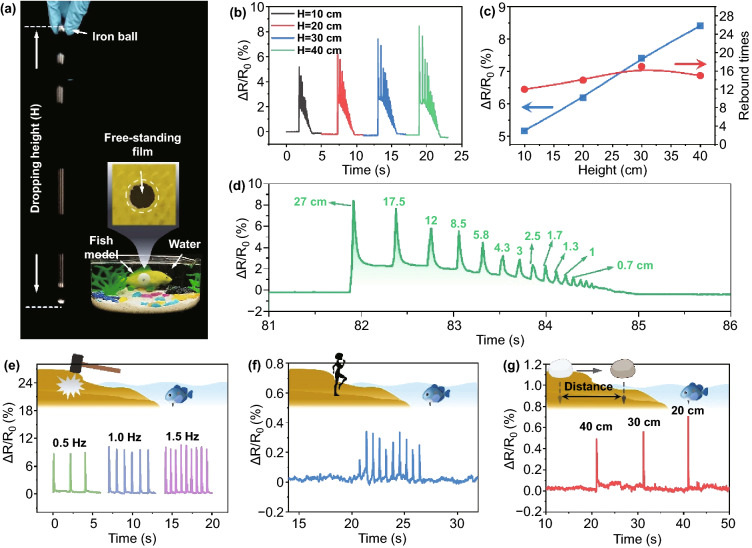
**a** Illustration of LMUS to sense the surrounding environment. **b** Δ*R*/*R*_0_ versus time curve of LUMS stimulated by the steel ball falling from different heights. **c** Δ*R*/*R*_0_ versus height curve of LUMS stimulated by steel ball (blue) and the rebound times of steel ball versus height curve (red). **d** Δ*R*/*R*_0_ versus time curve of LMUS during the rebounding process of steel ball falling from 40 cm. Δ*R*/*R*_0_ versus time curve of LMUS stimulated **e** by percussion at different frequencies; **f** by stomping; **g** by falling stones with a fixed height but different distances away from the water

It is well known that the fish can not only sense the water depth, but sensitively capture the signal over the water, to avoid some potential dangers. Similarly, LUMS was further employed to sense the mechanical stimuli from the external environment. As revealed in Fig. 5a, LUMS with a diameter of 10 mm was placed into water to perceive the environment signal. When an iron ball fell from the height of 10, 20, 30, and 40 cm, respectively (Fig. 5b), the device could clearly record the process that the ball hit and bounced off the table before coming to rest on the table. Moreover, the relative resistance was almost linearly related to the falling heights of the iron ball (Fig. 5c), and rebound times was precisely distinguished by LUMS, which was consistent with that counted by video (Fig. 5c). In addition, the time interval between each peak could be used to infer the bounce height of the iron ball (Fig. 5d). When LUMS was placed at a distance of 0, 2, and 4 cm below the water surface, the maximum relative resistance changes detected by the sensor were 0.05361, 0.11281, and 0.14284, respectively (Supplementary Fig. S23). But the response time of the sensor showed no significant difference with the change of underwater position. In general, the intensity of the relative resistance change generated by mechanical vibration increased as the depth of LUMS increased underwater. Besides, LUMS could detect signals with different frequencies produced by hitting the ground with a hammer (Fig. 5e) as well as the signal produced by stomping (Fig. 5f). Tests were also carried out to evaluate LUMS's perception of objects falling at different distances (20, 30, and 40 cm) away from the water (Fig. 5g), where the relative resistance decreased with the increase of the distance, demonstrating the good sensitivity. Therefore, our well-designed LUMS is expected to demonstrate potential applications in underwater and waterside monitoring.

**Fig. 6:**
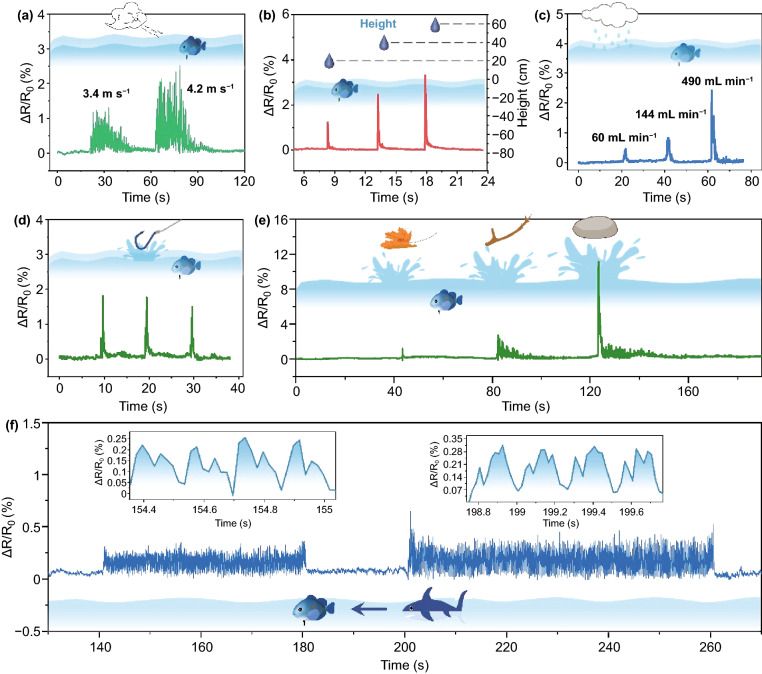
Δ*R*/*R*_0_ versus height curve of LUMS stimulated **a** by different wind speeds disturbing the liquid level and **b** by water droplet falling from different heights. Δ*R*/*R*_0_ versus height curve of LUMS stimulated **c** by different rainfalls; **d** by falling fishhook; **e** by a falling leaf, branch and stone; **f** by different fish swing amplitudes

To further simulate the excellent capability of underwater perception of fish, analogous to the function of the lateral line, LUMS can also sensitively capture the tiny and intense mechanical stimuli from and beneath the water surface. For instance, when the simulated wind with varied velocities was applied on the water surface, LUMS could rapidly perceive the fluctuation induced by the wind. Note that there are remarkable differences in the normalized resistance between the velocity of 3.4 and 4.2 m s^−1^ (Fig. 6a). In addition, a water droplet from different heights can also be sensitively detected. As shown in Fig. 6b, the droplet fallen from the height of 20, 40, 60 cm above the water surface could be effectively recognized. Moreover, in addition to the single water droplet, the raining condition was also simulated in our system. Figure 6c illustrates that LUMS can regularly respond to the simulated raining with different intensities, including 60, 144, and 490 mL min^−1^. More interestingly, similar to the fish, LUMS can actively perceive the water fluctuation induced by the fallen fishhook (Fig. 6d). In addition, some natural fallen objects such as leaf, branch, and stone, which led to tiny or drastic vibration/fluctuation of water surface, could be clearly detected (Fig. 6e). Furthermore, to efficiently tackle the potential danger beneath water surface, the sensor also needs to realize a sensitive perception of the underwater stimuli. As a proof of concept, a robotic shark swinging its tail was used to move around the sensor. As a result, LUMS could effectively monitor the distinct movement states of shark tails with low or large amplitudes, demonstrating significant potentials in applications of underwater monitoring (Fig. 6f).

## Conclusions

In summary, enlightened by the fish sensing system, an artificial fish lateral line enabled by the graphene-based thin film was designed to simultaneously detect wide-range water depth and sensitively perceive tiny vibration. The Janus-structured graphene/Ecoflex ultrathin film was endowed with conformal, elastic, conductive and deformable properties, which could adapt smoothly to the structured surface. When integrated to be a self-supported sensor, it can achieve an efficient and stable detection of water depth up to 1.8 m. Furthermore, tiny vibration (e.g. wind blowing, raining, leaf falling, and underwater attacking) from water surface or underwater can also be sensitively captured. The bioinspired Janus film-enabled underwater sensor shows significant potentials in the field of water depth detection and vibration perception in the water environment.

## Supplementary Information

Below is the link to the electronic supplementary material.

Supplementary file1 (PDF 989 KB)
