# Supplementary material for: Bioinspired Adaptive, Elastic, and Conductive Graphene Structured Thin-Films Achieving High-Efficiency Underwater Detection and Vibration Perception
Source: Nanomicro Lett. 2022 Feb 15;14:62. doi: 10.1007/s40820-022-00799-4 (PMC8844317; doi:10.1007/s40820-022-00799-4)
Supplement: Supplementary file 1 — Supplementary file1 (PDF 989 KB) [file 40820_2022_799_MOESM1_ESM.pdf]

Supporting Information for

Bioinspired Adaptive, Elastic and Conductive Graphene Structured Thin-Films Achieving High-Efficiency Underwater Detection and Vibration Perception

Qiling Wang[1, 2, #], Peng Xiao[1, 2, #, *], Wei Zhou[1, 2], Yun Liang[1, 2], Guangqiang Yin[1, 2], Qiu Yang[3], Shiao-Wei Kuo[4], and Tao Chen [1, 2, *]

[1]Key Laboratory of Marine Materials and Related Technologies, Zhejiang Key Laboratory of Marine Materials and Protective Technologies, Ningbo Institute of Materials Technology and Engineering, Chinese Academy of Sciences, Zhongguan West Road 1219, Ningbo 315201, P. R. China

[2]School of Chemical Sciences, University of Chinese Academy of Sciences, 19A Yuquan Road, Beijing 100049, P. R. China

[3]Ningbo New Material Testing and Evaluation Center Co., Ltd, Ningbo, 315000, P. R. China

[4]Department of Material and Optoelectronic Science, Center of Crystal Research, National Sun Yat-Sen University, Kaohsiung 804, Taiwan, P. R. China

[#]Qiling Wang and Peng Xiao contributed equally to this work

*Corresponding authors. E-mail: xiaopeng@nimte.ac.cn (Peng Xiao); tao.chen@nimte.ac.cn (Tao Chen)

Supplementary Figures

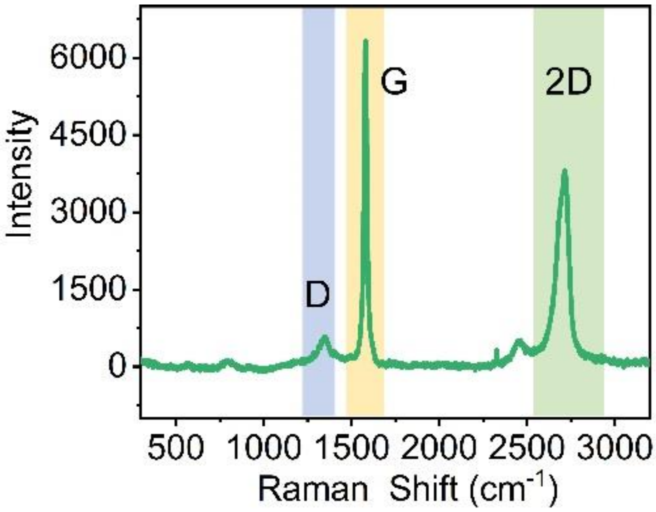

Fig. S1 Raman spectra of the pure graphene film

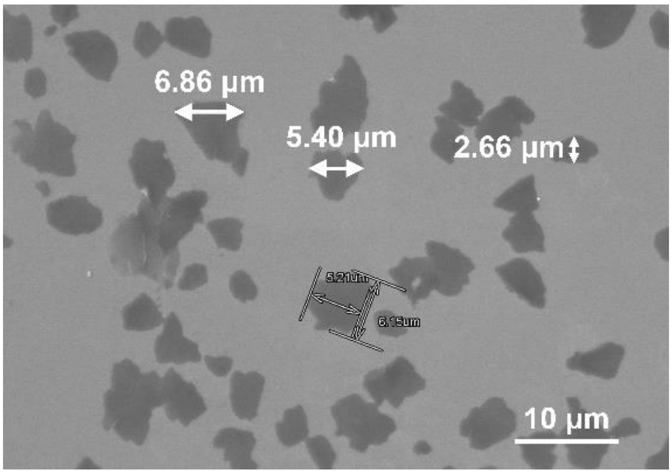

Fig. S2 SEM image of graphene flakes dispersed on silicon substrate

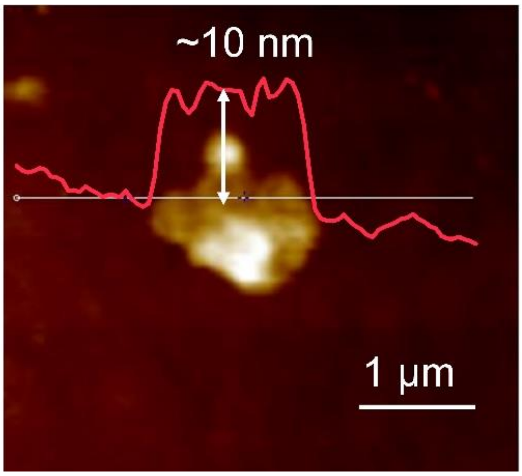

Fig. S3 The height of the graphene flake obtained by AFM

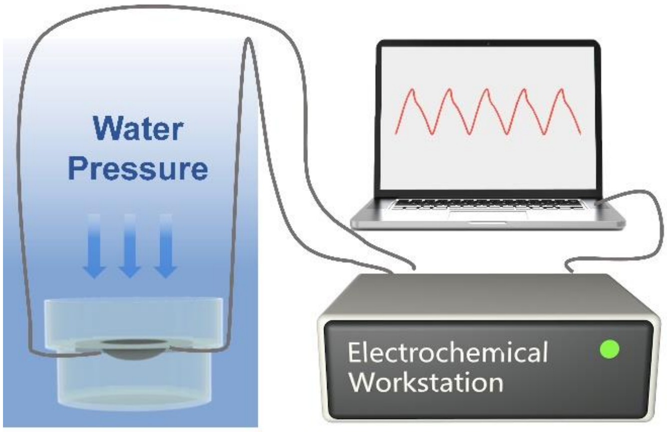

Fig. S4 The method to connect the sensor with the workstation and measurements

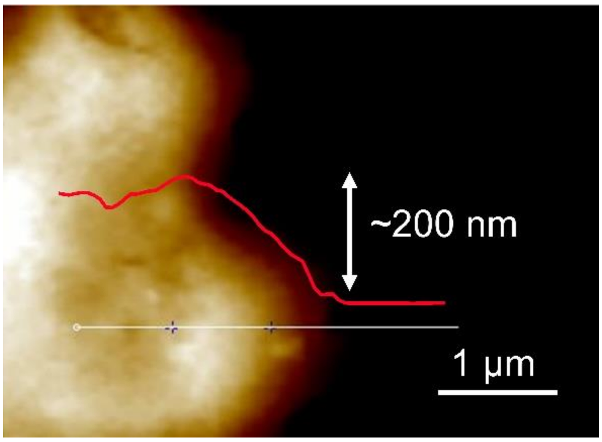

Fig. S5 The height of the graphene film obtained by AFM

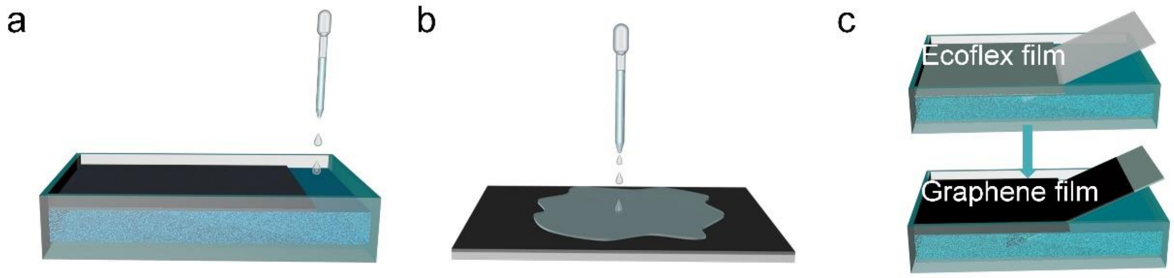

Fig. S6 Schematic diagram of interfacial asymmetric constructing method (**a**), casting method (**b**) and transferring method (**c**)

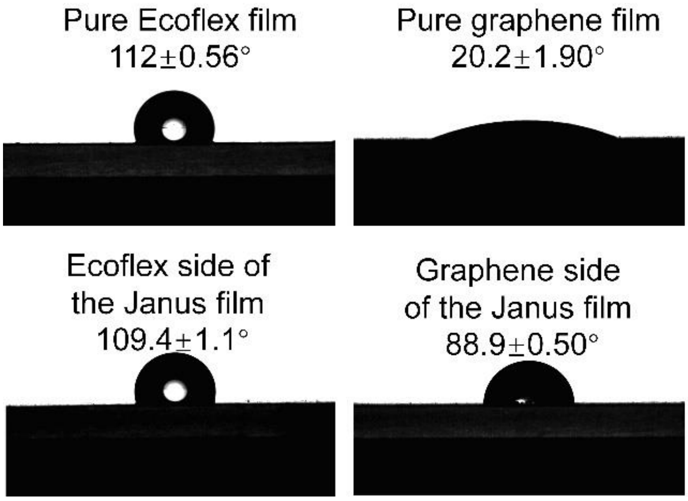

Fig. S7 The water contact angle of the pure Ecoflex film, the pure graphene film, and both sides surface of the Janus film

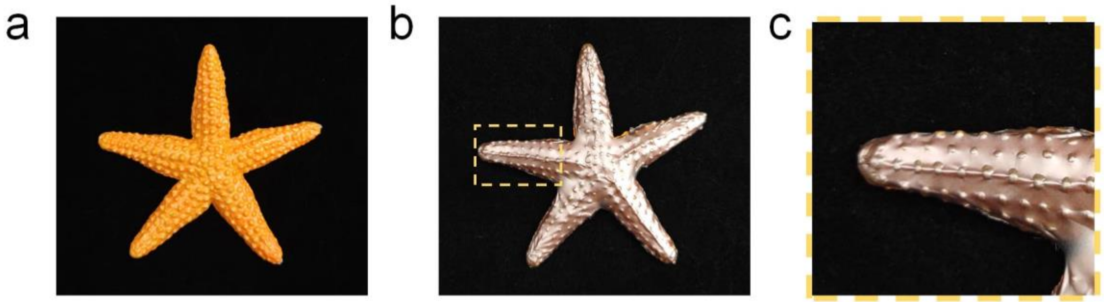

Fig. S8 The digital photos of the graphene/Ecoflex Janus film attached to the surface of the model starfish

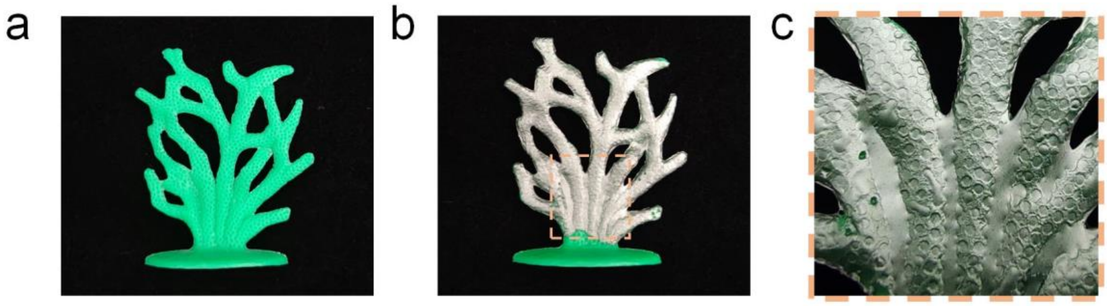

Fig. S9 The digital photos of the graphene/Ecoflex Janus film attached to the surface of the model seaweed

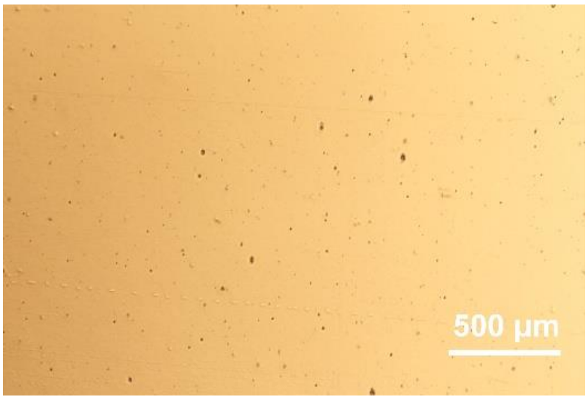

Fig. S10 Microscopic image of the tape surface after peeling off operation on the Janus film

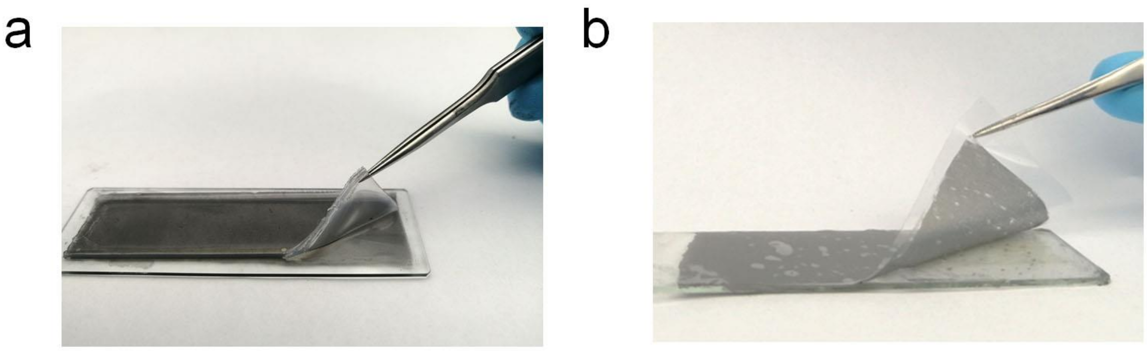

Fig. S11 The digital photos of casting film (**a**) and double-layer film (**b**) during peeling off operation

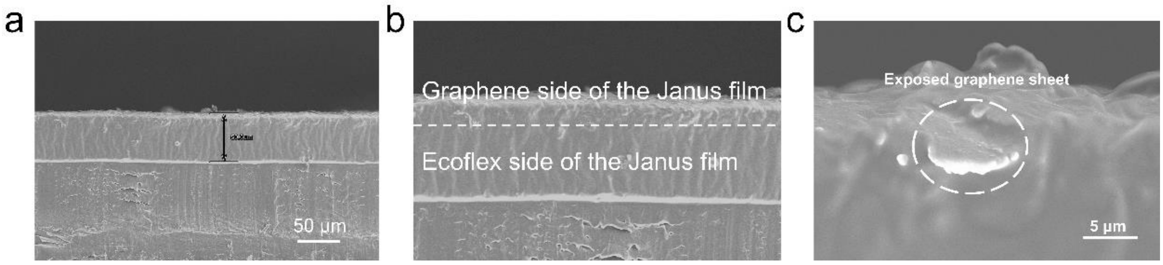

Fig. S12 SEM images of cross section of the graphene/Ecoflex Janus film (**a-b**). SEM image of cross section of the graphene/Ecoflex Janus film with exposed graphene sheet (**c**)

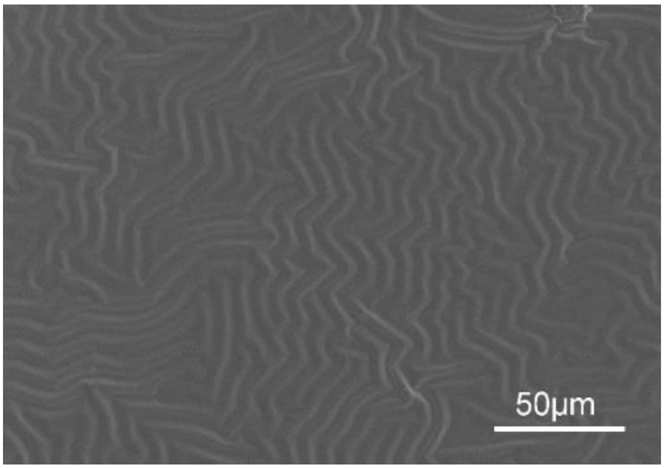

Fig. S13 SEM image of the Ecoflex side surface of the graphene/Ecoflex Janus film

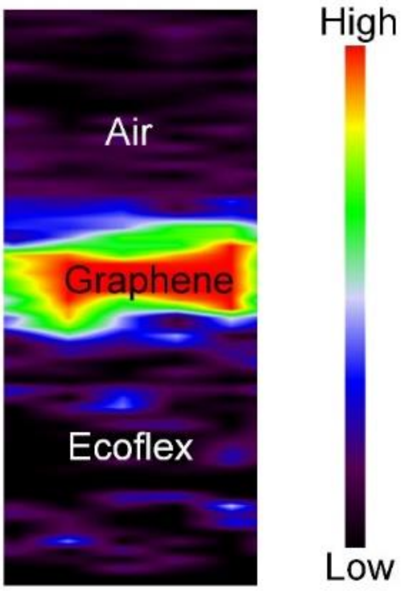

Fig. S14 Raman mapping image of the graphene/Ecoflex film

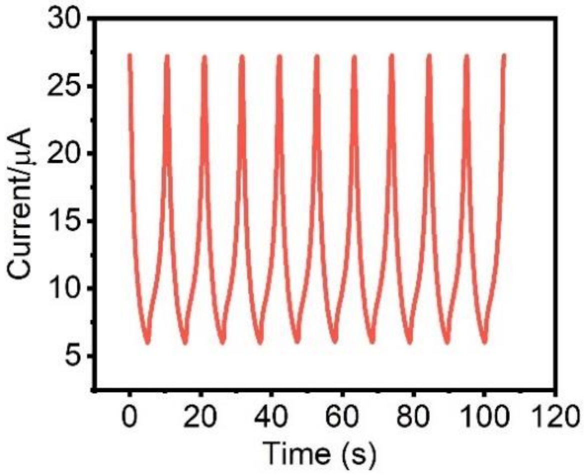

Fig. S15 Current variation of the graphene/Ecoflex Janus film under 10 times cyclic stretching from 0% to 20%

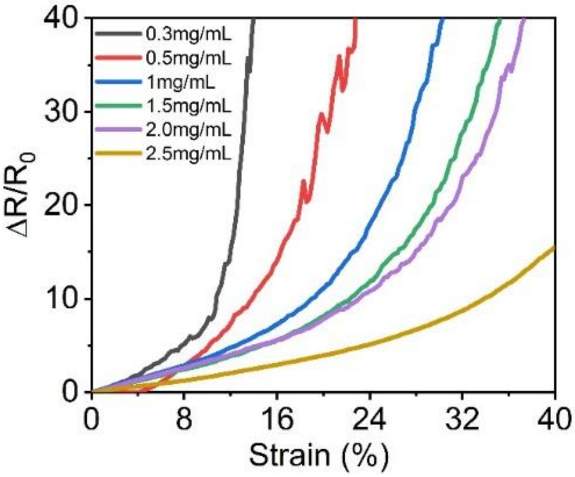

Fig. S16 The sensing performance of the Graphene/Ecoflex Janus film assembled by graphene dispersions of different concentrations

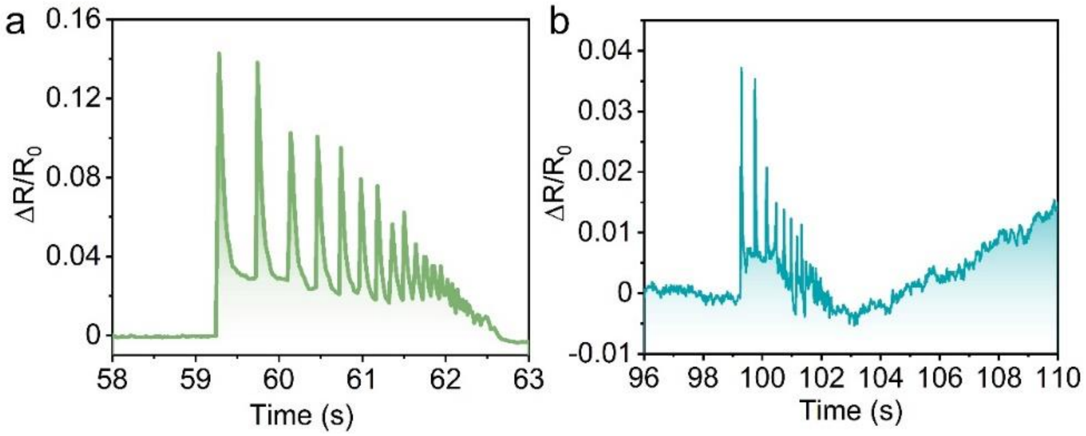

Fig. S17 a $\Delta R/R_0$ versus time curve of LMUS based on the Janus film placed at a distance of 4 cm from the water surface during the rebounding process of steel ball falling from 40 cm. b $\Delta R/R_0$ versus time curve of LMUS based on the encapsulated Janus film placed at a distance of 4 cm from the water surface during the rebounding process of steel ball falling from 40 cm

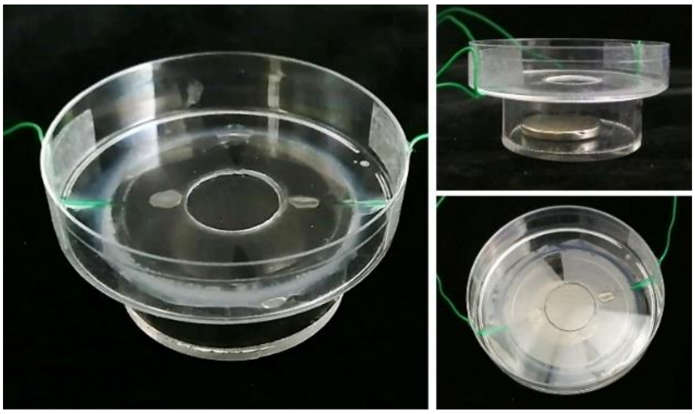

Fig. S18 The digital photos of the lateral line imitating underwater sensor from different angles

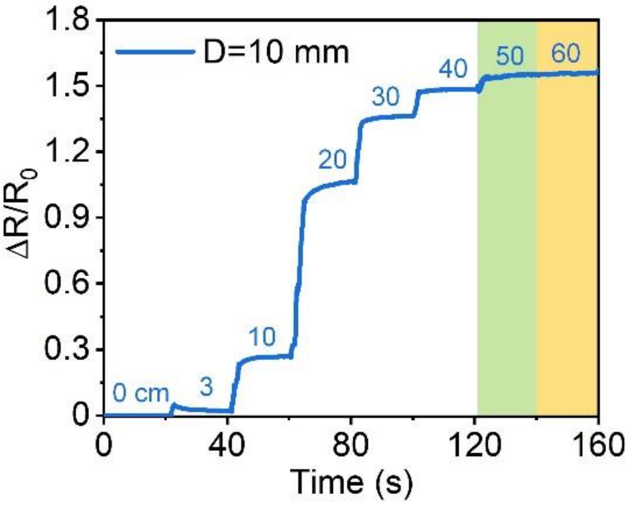

Fig. S19 $\Delta R/R_0$ versus time curve of the graphene/Ecoflex Janus film with a diameter of 10 mm at different depths

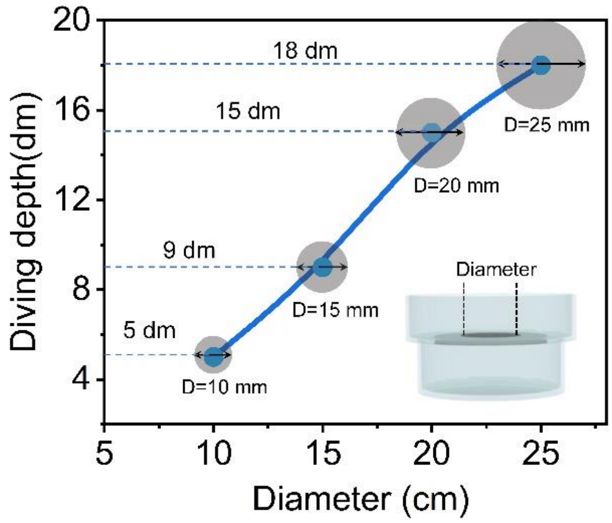

Fig. S20 The curve of the maximum measurable depth as a function of the film diameters

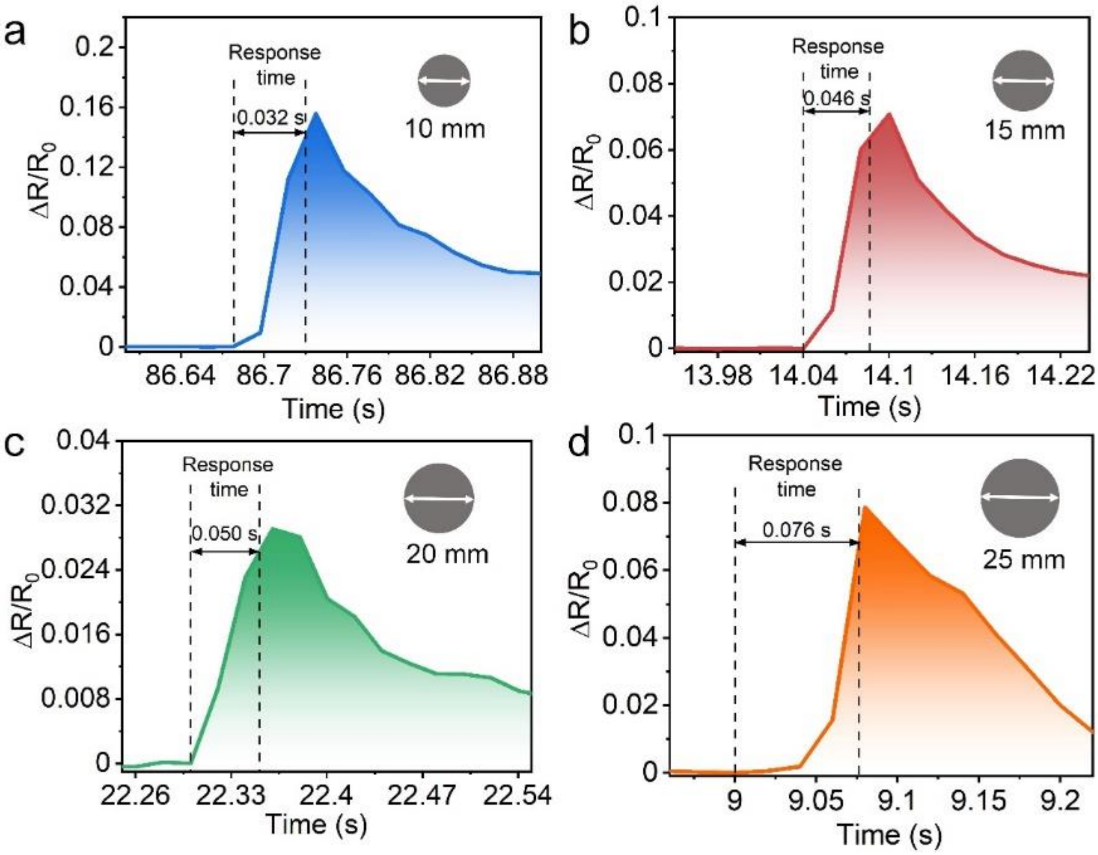

Fig. S21 Measurement of response time from the relative current variation curve of the sensor with a film diameter of (**a**)10 mm, (**b**) 15 mm, (**c**) 20 mm, (**d**) 25 mm

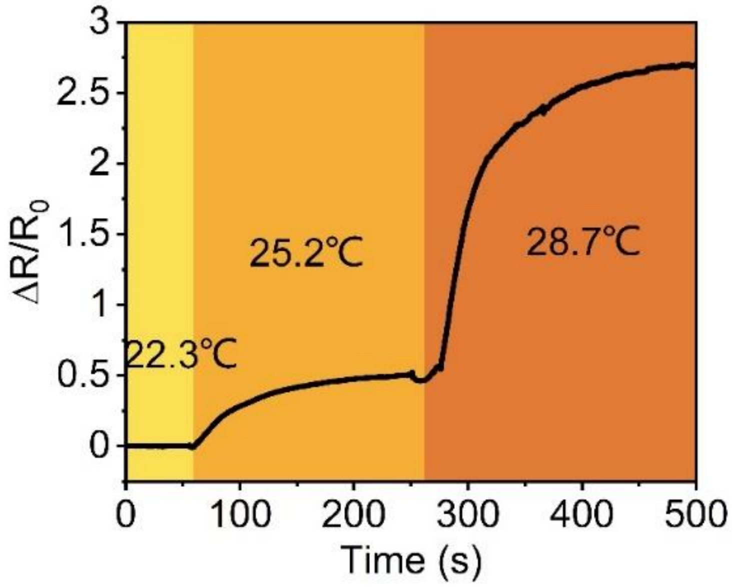

Fig. S22 $\Delta R/R_0$ versus time curve of the graphene/Ecoflex Janus film with a diameterof 10 mm at different water temperature

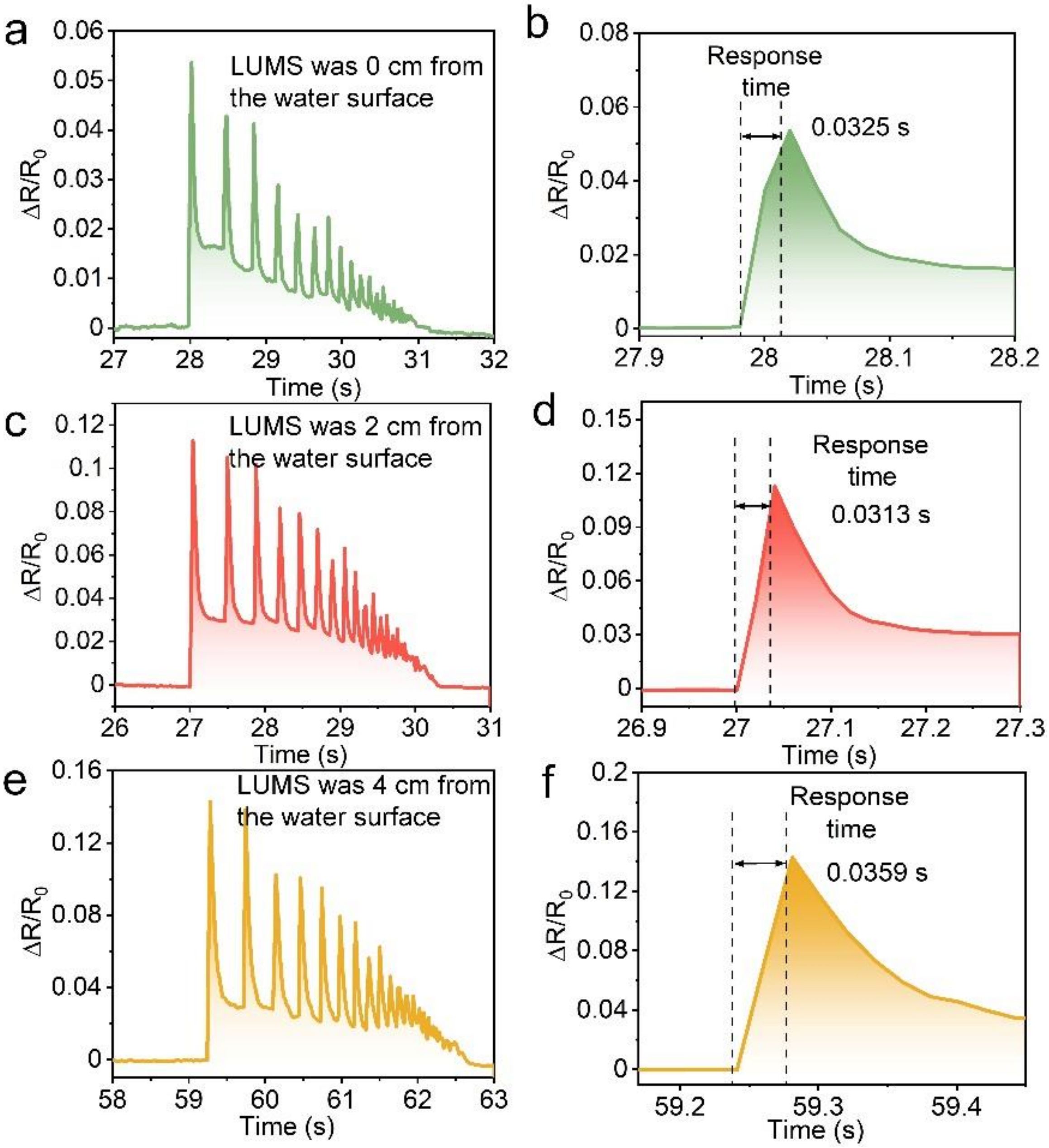

Fig. S23 $\Delta R/R_0$ versus time curve of LMUS based on the Janus film placed at a distance of 0 cm from the water surface during the rebounding process of steel ball falling from 40 cm (**a**) and corresponding response time (**b**). $\Delta R/R_0$ versus time curve of LMUS based on the Janus film placed at a distance of 2 cm from the water surface during the rebounding process of steel ball falling from 40 cm (**c**) and corresponding response time (**d**). $\Delta R/R_0$ versus time curve of LMUS based on the Janus film placed at a distance of 4 cm from the water surface during the rebounding process of steel ball falling from 40 cm (**e**) and corresponding response time (**f**)
